# Putative autoantibodies in the cerebrospinal fluid of Alzheimer’s disease patients

**DOI:** 10.12688/f1000research.21140.1

**Published:** 2019-11-11

**Authors:** Bryant Lim, Magda Tsolaki, Ihor Batruch, Anna Anastasiou, Antonis Frontistis, Ioannis Prassas, Eleftherios P. Diamandis

**Affiliations:** 1 Department of Laboratory Medicine and Pathobiology, University of Toronto, Toronto, Ontario, Canada; 2 1st Department of Neurology, Medical School, Aristotle University of Thessaloniki, Thessaloniki, Greece; 3 Department of Pathology and Laboratory Medicine, Mount Sinai Hospital, Toronto, Ontario, Canada; 4 Department of Clinical Biochemistry, University Health Network, Toronto, Ontario, Canada

**Keywords:** Alzheimer’s disease, Parkinson’s disease, cerebrospinal fluid, autoantibodies, immuno-mass spectrometry, biomarkers, glia-derived nexin, actin-interacting protein, quinone oxidoreductase, sushi repeat-containing protein, metalloproteinase inhibitor 2, inositol 1, 4, 5-triphosphate receptor type 1, sarco/endoplasmic reticulum calcium ATPase 2

## Abstract

**Background:** Recent efforts have described an immunogenic component to the pathobiology of Alzheimer’s disease (AD) and Parkinson’s disease (PD). However, current methods of studying fluid autoantibodies, such as enzyme-linked immunosorbent assays and immunohistochemistry, are hypothesis-driven and not optimal for discovering new autoantibody biomarkers by proteome-wide screening. Recently, we developed a general mass spectrometry-based approach to identify tissue-specific autoantibodies in serum, at a proteome-wide level. In this study, we adapted the method to explore novel autoantibody biomarkers in the cerebrospinal fluid (CSF) of AD and PD patients.

**Methods:** CSF samples were obtained from 10 headache control individuals, 10 AD patients and 10 PD patients. Antibodies present in the CSF were isolated by immobilization to protein-G magnetic beads. These antibodies were incubated with a brain tissue extract, prepared from frontal cortex, pons, cerebellum and brain stem. Protein antigens captured by the protein-G magnetic bead-bound antibodies were digested with trypsin and analyzed using mass spectrometry. Autoantibody candidates were selected by 1) detection in one or less individuals of the control group and 2) identification in at least half of the patient groups.

**Results: **There were 16 putative autoantibody biomarkers selected from the AD group. Glia-derived nexin autoantibody was detected in eight of ten AD patients and was absent in the control group. Other AD pathology-related targets were also identified, such as actin-interaction protein, quinone oxidoreductase, sushi repeat-containing protein, metalloproteinase inhibitor 2, IP3 receptor 1 and sarcoplasmic/endoplasmic reticulum calcium ATPase 2. An additional eleven autoantibody targets were also identified in the present experiment, although their link to AD is not clear. No autoantibodies in the PD group satisfied our selection criteria.

**Conclusion: **Our unbiased mass spectrometry method was able to detect new putative CSF autoantibody biomarkers of AD. Further investigation into the involvement of humoral autoimmunity in AD and PD pathobiology may be warranted.

## Introduction

Significant efforts have been made on advancing diagnostic protein biomarkers of Alzheimer’s (AD) and Parkinson’s (PD) disease, the most common forms of neurodegenerative diseases. These discoveries inform the underlying pathobiology and innovative therapeutics for AD and PD ^1, 2^. Though the causes of neurodegeneration are largely unknown, recent research hints to an autoimmune component to these diseases ^3^.

The notion of immune privilege of the central nervous system (CNS) has been challenged by studies revealing functional lymphatic systems that drain cerebrospinal fluid (CSF) to peripheral lymph nodes, prompting re-evaluation of the role of adaptive immunity in neurodegenerative diseases ^4^. In studies linking autoimmune mechanisms to AD, D’Andrea observed immunoglobulin G (IgG)-specific neuron degeneration through a classical complement pathway mediated by microglia in AD post-mortem brains ^5, 6^. In PD, post-mortem studies of brain tissue showed IgG binding and alterations in CD4 ^+^ and CD8 ^+^ T cell levels in proximity to dopamine neurons, suggesting a potential autoimmune involvement in PD progression ^7^. Changes in brain-related autoantibody levels in CSF and serum of AD and PD patients have also been identified. The targeted self-antigens include pathology-related protein aggregates, neurotransmitters, surface receptors, glial markers, lipids and cellular enzymes ^8, 9^.

Currently, experimental techniques to identify biofluid autoantibodies are limited. The primary methods to quantify autoantibodies are radiobinding assays, immunohistochemistry, enzyme-linked immunosorbent assays (ELISA), bead-based assays and protein microarrays ^10^. Most of these tools, however, require an *a priori* hypothesis, and are limited to single-target profiling. High-throughput methods such as human protein microarrays have yielded novel autoantibody markers, but they are costly and require recombinant protein availability and optimization ^10^. Recently, our laboratory developed an unsupervised mass-spectrometry-based protocol using a data-dependent acquisition approach to identify tissue-specific autoantibodies ^11^. Here, we applied this protocol in a preliminary study to identify novel brain-specific autoantibodies in the CSF of AD and PD, using a cohort of 10 headache control individuals, 10 AD and 10 PD patients.

## Methods

### Sample collection

CSF was retrospectively collected from a total of 30 individuals between 2014 and 2019 at the memory and dementia clinic of the 1st and 3rd Department of Neurology, AHEPA and “G. Papanicolaou” Hospitals, School of Medicine, Aristotle University of Thessaloniki, Greece. The study was approved with written informed consent from study individuals and by the Greek Alzheimer Association and Related Disorders (GAARD) scientific and ethics committees, and the Institutional Review Board of the University of Toronto.

The study participants included 10 control individuals with headache, 10 patients with AD and 10 patients with PD. Clinical diagnosis of probable AD was made based on the NINCDS/ADRDA criteria for probable AD with a threshold cut-off for AD at a Mini-Mental State Examination (MMSE) score of 26 ^12^. Clinical diagnosis of PD was made based on the modified Hoehn and Yahr (H-Y) scale ^13^. Functional Rating Scale for Symptoms of Dementia (FRSSD) was also measured to assess the impact of dementia on patients’ daily activities.

Following confirmation of diagnosis, CSF samples were collected by lumbar puncture in the morning, centrifuged to remove cellular components and stored at -80°C polypropylene tubes. The samples were then shipped to the Lunenfeld Tanenbaum Research Institute, Mount Sinai Hospital, Toronto, Canada and stored at -80°C until further processing.

### Tissue protein extraction

Total protein was extracted from four regions of the brain: frontal cortex, pons, cerebellum and brain stem. Each tissue was pulverized in liquid nitrogen using a mortar and pestle. The pulverized tissue was further digested with 0.2% RapiGest SF Surfactant (Waters, Milford, MA, USA) in 50 mM ammonium bicarbonate (ABC) for 30 min on ice, while vortexing every 2–5 min. The homogenate was sonicated on ice for three times, 15 s each, and centrifuged at 15,000 g for 20 min at 4°C. The resulting pellet containing debris and insoluble contaminants was removed. Pierce bicinchoninic acid assay (Thermo Fisher Scientific, San Jose, California) was performed to determine total protein concentration. Fractions from each brain region were pooled in equal parts (in terms of total protein contribution).

### Immunoprecipitation on protein-G magnetic beads and on-bead trypsin digestion

The experimental protocol has been described elsewhere ^11^. Briefly, 50 µL of 10% w/v Protein-G Mag Sepharose Xtra magnetic beads (GE Healthcare) medium slurry was resuspended by vortexing and added to a microcentrifuge tube. The microcentrifuge tube was placed in a magnetic separator, and the storage solution was removed. The magnetic beads were washed with 500 µL PBS. CSF samples were spiked with 100 ng of human kallikrein 6 (HK6) mouse monoclonal antibody, purified in-house with high sensitivity and specificity ^14^, as a positive control and added to the magnetic beads. PBS was added to the mixture to reach a final volume of 300 µL. IgG from the CSF was bound to the beads during a 30 min incubation with gentle rotation. After two washes with 500 µL PBS, 100 µg of the pooled brain lysate was added to the beads, followed by a 2-hour incubation with gentle rotation. Following incubation, the beads were washed three times with 500 µL PBS 0.05% Tween 20, and subsequently washed three more times with 500 µL PBS. The beads were reconstituted in 100 µL PBS.

The reconstituted beads, along with the captured antibodies and antigens, were reduced by adding 100 mM dithiothreitol (DTT) to a final concentration of 5 mM, and incubated at 56°C for 40 min. For alkylation, 500 mM iodoacetamide (IAA) was added to a final concentration of 15 mM and incubated for 30 minutes in the dark with gentle shaking. For digestion, trypsin was added to each sample in a 1:50 enzyme to substrate ratio and incubated at 37°C overnight with gentle shaking. The supernatant was collected using the magnetic separator, and formic acid was added to a final concertation of 1%, reaching a pH of 2, to stop the reaction.

### Mass spectrometry analysis of immunoprecipitated brain-specific antigens

Peptides were purified by extraction using OMIX C18 tips (Agilent Technologies, Santa Clara, CA), eluted with 3 µL acetonitrile buffer solution (0.1% formic acid in 65% acetonitrile) supplemented with 57 µL of 0.1% formic acid. Using an auto-sampler, 18 µL of sample, run in technical duplicates, was injected from a 96-well plate into a C18 Acclaim PepMap 100 (75 µm x 2 cm, C18 3 µm bead, 100 Å pore size) trap column (Thermo Fisher Scientific, San Jose, California) and peptides were eluted into a 50 cm analytical column (PepMap RSLC C18, 75 μm ID, 2 μm bead, 100 Å pore, ES803, Thermo Fisher Scientific). The liquid chromatography, EASY-nLC 1200 system (Thermo Fisher Scientific), was coupled online to a Q Exactive HF-X (Thermo Fisher Scientific) mass spectrometer with the EASY-Spray ionization source (Thermo Fisher Scientific) with a spray voltage of 2 kV and capillary temperature at 320°C. The 60-minute liquid chromatography (LC) was applied at a flow rate of 250 nl/min with an increasing concentration of buffer D (0.1% formic acid in 95% acetonitrile). In a 60-min data-dependent acquisition (DDA) mode, full MS1 scan was acquired from 400 to 1500 *m/z* at a resolution of 60,000 in profile mode, followed by MS2 scans of the top 28 parent ions at a resolution of 15,000. Dynamic exclusion was set to 20 s, and 1+ and 6+ or more charge state ions were excluded from MS2 fragmentation. MS method parameters were detailed previously ^11^.

### Data analysis

Raw files were uploaded into the Proteome Discoverer v.1.4 (Thermo Fisher Scientific) and searched with Sequest HT search engine against the Human 5640 Swiss-Prot protein database (January 2018) ( MaxQuant is an open-source alternative to Proteome Discoverer). The search parameters included: trypsin enzyme with two maximum missed cleavages, cysteine carbamidomethylation as a static modification, precursor mass tolerance of 7 ppm, fragment mass tolerance of 0.02 Da, methionine oxidation as a dynamic modification, 1% false-discovery rate (FDR) at the peptide and protein level using the Percolator node.

Abundant serum proteins that may bind non-specifically to the beads, including hemoglobin, haptoglobin, hemopexin, immunoglobins, keratins, apolipoproteins, serum albumin and complement, were removed from the initial candidate selection. Candidate autoantibody biomarkers were identified by antigens that were 1) absent in the patient control group, defined as identification in a maximum of one out of the 10 control individuals and 2) identified in the patient group, defined as presence in at least half of the patients (5 out of 10).

Statistical analyses for clinical descriptions were performed using GraphPad Prism v. 6.0e. A *p*-value <0.05 was considered significant. Chi-square test (overall and pairwise) was used to compare categorical demographic characteristics of the three groups. Non-parametric Kruskal-Wallis test by ranks was used to compare characteristics on a continuous scale. Dunn’s multiple comparison test was applied for pairwise comparisons.

## Results

### Patient demographics

Patient descriptions are shown in Table 1 and *Underlying data* ^15^. There were no significant differences in the proportion of males and females. Age was significantly different between groups ( *p* = 0.0002), and pairwise comparisons revealed lower age in the control group than both AD ( *p* = 0.0005) and PD ( *p* = 0.0025) patient groups. MMSE score was significantly different between groups (<0.0001), and multiple comparisons revealed that AD ( *p* < 0.0001) and PD ( *p* = 0.0188) groups had lower MMSE scores than the control group. The median (interquartile range) of the H-Y score in the PD group was 2.0 (1.5-2.8). FRSSD was not significantly different between AD and PD groups.

**Table 1.** Patient cohort characteristics.

Characteristics	Headache control	Alzheimer’s disease	Parkinson’s disease	*p*-value
Participants, n	10	10	10	
Sex-female, n (%)	6 (60)	6 (60)	4 (40)	0.5853
Age ^a^	40 (38.3, 49.3)	76.5 (73.3, 80.0)	74.0 (69.0, 79.0)	0.0002
MMSE score ^a, b^	28 (28, 28)	18 (14, 20)	22 (19, 25)	<0.0001
H-Y score ^a, c^			2 (1.5, 2.8)	
FRSSD ^a, d^		10 (9, 11)	12 (6, 17)	0.9764

^a^Expressed as median (25 ^th^, 75 ^th^ percentile) ^b^Mini-mental status examination ^c^Hoehn and Yahr score ^d^Functional Rating Scale for Symptoms of Dementia

### Tissue lysate protein concentrations

The total protein content from human brain regions, frontal cortex, pons, cerebellum and brain stems, ranged from 1.7 mg/mL to 5.3 mg/mL ( *Extended Data*: Supplementary Table 1 ^16^).

### Cerebrospinal fluid self-antigen identification

**Figure 1.**
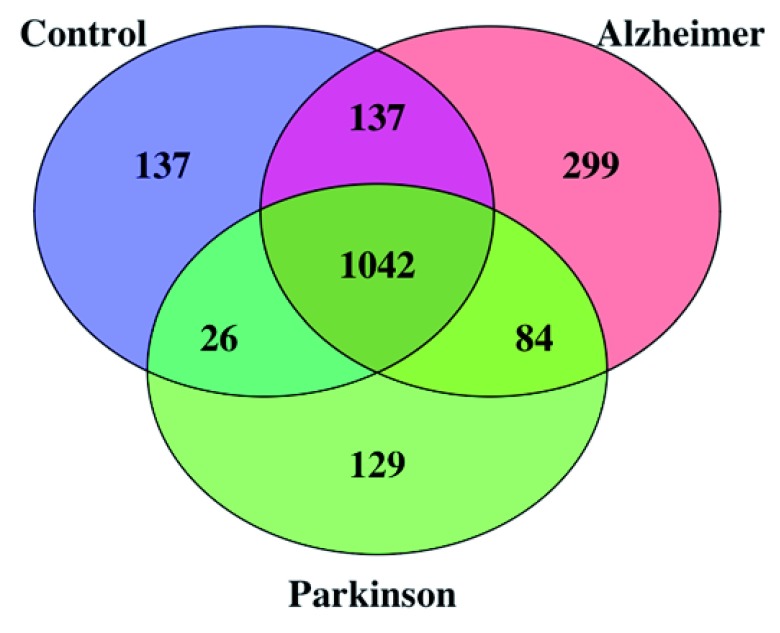
Venn diagram of proteins identified in CSF samples from the control, Alzheimer’s disease (AD) and Parkinson’s disease (PD) groups. Total number of identified antigens in all samples was 1854 with 1042 (56%) common antigens in all groups. Number of identified antigens in control, AD and PD groups were 1342, 1562 and 1281 respectively.

Antibodies from the CSF, bound to protein-G beads, captured putative cognate antigens from the brain tissue-mix. Mass spectrometry identification of these cognate autoantigens infer presence of brain-specific autoantibodies from the CSF. Using a 1% FDR for peptide identification, the number of antigens detected in each individual CSF ranged from 461 to 1192, amounting to 1508, 1754, 1452 total antigens identified in the control, AD and PD groups, respectively. After removal of abundant serum proteins, 1342, 1562 and 1281 antigens were remaining in control, AD and PD respectively. The number of antigens that were uniquely found in control, AD and PD groups was 137, 299 and 129, respectively. A Venn diagram of the putative CSF autoantibody-bound antigens detected in each group, after removal of abundant serum antigens is shown in Figure 1. The positive control, human kallikrein 6, (hK6), was abundantly identified in all samples, with nine to 13 unique peptides ( *Extended Data*: Supplementary Table 2 ^16^). See *Underlying data* ^15^ for details of all.

### Candidate selection of AD and PD patient groups

In total, 16 putative autoantibodies fulfilled our aforementioned selection criteria in the AD group. No candidates fulfilled the criteria in the PD group. The candidates, along with the number of unique peptides identified for each antigen, are summarized in Table 2. More details on the identity of each identified peptide per antigen, are shown in *Extended Data*: Supplementary Table 2 ^16^.

**Table 2.** Putative cerebrospinal fluid (CSF) autoantibody biomarkers of Alzheimer’s disease (AD), selected based on 1) identification in half or more of the patient group and 2) present in one or less individuals of the control group.

	Control individuals			AD group			PD group		
Protein name	Number of individuals identified	Total unique peptides	Mean unique peptides	Number of patients identified	Total unique peptides	Mean unique peptides	Number of patients identified	Total unique peptides	Mean unique peptides
Glia-derived nexin (SERPINE2)	0	0	0	8	7	2.3	3	2	1
Fibromodulin (FMOD)	0	0	0	6	4	1.5	0	0	0
Quinone oxidoreductase (NQO1)	0	0	0	5	1	1	3	1	1
Cathepsin F (CTSF)	1	1	1	6	5	2.3	1	1	1
Cadherin-13 (CDH13)	1	1	1	6	5	2	1	1	1
Phospholipase D4 (PLD4)	1	1	1	6	1	1	3	1	1
Inositol 1,4,5- triphosphate receptor type 1 (ITPR1)	1	1	1	5	6	1.4	0	0	0
Sushi repeat- containing protein (SRPX)	1	1	1	5	5	1.8	3	1	1
Sarco/endoplasmic reticulum calcium ATPase 2 (ATP2A2)	1	1	1	5	5	1.2	2	1	1
Oligodendrocyte- myelin glycoprotein (OMG)	1	1	1	5	4	1.4	3	1	1
4F2 cell-surface antigen heavy chain (SLC3A2)	1	1	1	5	4	1.2	2	1	1
WD repeat- containing protein 1 (WDR1)	1	1	1	5	3	1.4	2	2	1.5
Isoaspartyl peptidase (ASRGL1)	1	1	1	5	2	1.4	2	1	1
Heterogeneous nuclear ribonucleoprotein H (hnRNP H)	1	1	1	5	2	1.2	1	1	1
Metalloproteinase inhibitor 2 (TIMP-2)	1	1	1	5	2	1.2	1	1	1
Cerebellin-3 (CBLN3)	1	1	1	5	2	1	0	0	0

For AD, three autoantibodies against brain-specific antigens, glia-derived nexin (SERPINE2), fibromodulin (FMOD) and quinone oxidoreductase (NQO1), were absent in CSF of all patients in the control group and were present in eight, six and five patients with AD, respectively ( Table 2). SERPINE2 was identified with an average of 2.3 unique peptides in each individual ( *Extended Data*: Supplementary Table 2 ^16^), totaling seven unique peptides in the whole patient group. FMOD was identified with an average of 1.5 unique peptides per patient with a total of 4 peptides in the whole patient group. Finally, NQO1 was identified in five patients with one unique (the same) peptide per patient ( *Extended Data*: Supplementary Table 2 ^16^).

A further 13 putative autoantibodies against brain-specific antigens were found in one out of ten control individuals and at least half of the AD patients. In all cases in the control group, the antigen was identified using only one unique peptide ( Table 2 and *Extended Data*: Supplementary Table 2 ^16^). Autoantibodies against cathepsin F (CTSF), cadherin-13 (CDH13), and phospholipase D4 (PLD4) were identified in six AD patients, with an average of 2.3, 2 and 1 unique peptide per patient, respectively ( Table 2 and *Extended Data*: Supplementary Table 2 ^16^). The remaining candidates were identified in five AD patients ( Table 2). This included inositol 1,4,5-triphosphate receptor type 1 (ITPR1, or IP3 receptor), sushi repeat-containing protein (SRPX), isoaspartyl peptidase (ASRGL1), heterogeneous nuclear ribonucleoprotein H (hnRNP H), cerebellin-3 (CBLN3), oligodendrocyte-myelin glycoprotein (OMG), metalloproteinase inhibitor 2 (TIMP-2), WD repeat-containing protein 1 (WDR1, or AIP1), 4F2 cell-surface antigen heavy chain (SLC3A2) and sarco/endoplasmic reticulum calcium ATPase 2 (ATP2A2, or SERCA2). The number of unique peptides detected for each antigen ranged from 1 to 1.8 ( Table 2 and *Extended Data*: Supplementary Table 2 ^16^).

## Discussion

The relevance of autoimmune mechanisms in AD and PD pathobiology is not well understood. In the present study, we adapted an in-house-designed novel mass-spectrometry-based protocol to explore brain-specific autoantibody biomarkers in the CSF of AD and PD patients ^11^. Presence of autoantibodies is inferred by identification of their cognate antigens. To our knowledge, this is one of the first studies using a non-biased mass spectrometry approach for autoantibody discovery in CSF of AD and PD patients.

Putative AD and PD-relevant self-antigens were defined by 1) identification in one or less individuals in the patient control group (n=10) and 2) presence in at least half of the patient group (n=10 each). Using these preset selection criteria, we identified 16 putative brain-specific autoantibodies related to AD. No candidates were identified for PD.

Presence of autoantibodies against SERPINE2 was detected in eight of the ten AD patients with an average of 2.3 unique peptides for the antigen; no peptides were identified in any individuals from the control group. SERPINE2, one of several members in the SERPIN superfamily, is a serine protease inhibitor constitutively secreted by glial cells, and plays a key role in synaptic plasticity for developing and adult CNS ^17– 19^. In AD pathology, post-mortem patients show that SERPINE2 levels are related to tau-positive dystrophic neurites and amyloid protein processing in the hippocampus ^20, 21^. Furthermore, SERPINE2 is a potent regulator of thrombin, a proximate proinflammatory mediator of blood brain barrier dysfunction implicated in AD ^22, 23^. Presence of autoantibodies targeting SERPINE2 may reflect a biological relationship between AD pathogenesis and SERPINE2. Other autoantibodies targeting brain antigens implicated in AD pathology, including WDR1, NQO1, SRPX, TIMP-2, ITPR1 and ATP2A2, were also identified. These proteins are involved in a variety of neurological processes related to AD such as mediating amyloid-beta induced cytotoxicity ^24, 25^, antioxidant activity ^26– 29^, amyloid plaque co-accumulation ^30^, blocking of Aβ-induced release of lactate dehydrogenase ^31^, maintaining age-related neuronal plasticity ^32^, and mediating presenilin-controlled calcium ion homeostasis ^33– 36^. Autoantibodies against FMOD, CDH13, CTSF, PLD4, SRPX, ASRGL1, hnRNP H, CBLN3, OMG and SLC3A2 were also identified in the present study, although their link to AD is not well understood.

Interestingly, no autoantibody biomarker candidates were identified in PD using the same selection criteria. Whether this is due to the insensitivity of the method or to the lesser involvement of autoimmunity in PD cannot be determined.

There are several limitations with this study. The significantly younger control group could be a confounding factor, leading to lower abundance of autoantibodies in the control group ^37^. The study comprises a small sample size, and therefore candidates would require further verification in a larger cohort using recombinant proteins or orthogonal methods, such as ELISA. Finally, this exploratory method may identify autoantibodies of unknown significance, and is unable to establish a causal link between the identified autoantibodies and disease processes. Functional studies must be conducted to delineate the roles of each autoantibody in disease pathology.

The role of humoral immunity in the pathogenesis of AD and PD remains a controversial topic. However, given that over 99% of compounds entering phase I trials for AD never reach approval and the mounting evidence of the multi-faceted complexity of AD pathology, innovative research perspectives and technologies are necessary to explore its pathobiology from alternative angles ^38^. Biomarkers identified from these approaches could subsequently inform our understanding of the underlying biology and potential therapeutics. In the present study, we adapted a novel unsupervised proteomic approach to detect potential immunogenic components of AD and PD, and identified promising autoantibody biomarkers of AD. Future studies focusing on an autoimmune pathogenesis of AD, but not PD, are warranted.

## Data availability

### Underlying data

Harvard Dataverse: Putative autoantibodies in the cerebrospinal fluid of Alzheimer’s disease patients. https://doi.org/10.7910/DVN/DYMEQR ^15^.

This project contains the following underlying data:

- Patient descriptives.txt (clinical data, including demographic information and clinical presentation data for each enrolled patient).
- Shotgun mass spectrometry for brain specific autoantibodies.tab (Raw mass spectrometry data showing identified antigens).

### Extended data

Harvard Dataverse: Extended data for "Putative autoantibodies in the cerebrospinal fluid of Alzheimer’s disease patients" https://doi.org/10.7910/DVN/JJW0LG ^16^.

This project contains the following extended data:

Supplementary Table 1 (Total protein concentration in each human brain tissue extract as determined by Pierce BCA Protein Assay).
- Supplementary Table 2 (Brain-specific self-antigens and the number of associated peptides used to identify the protein in each sample. In the expanded spreadsheet, "I" indicates that the peptide was identified in the patient CSF sample, while blank cells indicate absence).
- Data are available under the terms of the Creative Commons Zero “No rights reserved” data waiver (CC0 1.0 Public domain dedication).
