# Supplementary material for: Putative autoantibodies in the cerebrospinal fluid of Alzheimer’s disease patients
Source: F1000Res. 2019 Nov 11;8:1900. [Version 1] doi: 10.12688/f1000research.21140.1 (PMC7008601; doi:10.12688/f1000research.21140.1)
Supplement: Peer review history

# Peer review: Reviewer response for version 1

**DOI:** 10.5256/f1000research.23271.r56422

**Published:** 2020-10-05

**Authors:** Mohd M. Khan

**Affiliations:** 1 Booz Allen Hamilton, McLean, VA, USA

Each year about 10 million new cases of dementia are reported worldwide. In the US alone, Alzheimer's disease (AD) is the 6 ^th^ leading cause of death as it affects >5 million Americans. AD is generally diagnosed by conducting tests to assess memory impairment as well as using brain imaging such as Magnetic resonance imaging (MRI), Computerized tomography (CT), and Positron Emission Tomography (PET) based tests. Numerous studies have proposed potential use of biomarkers (e.g., beta-amyloid and tau levels in cerebrospinal fluid (CSF) and brain changes detectable by imaging), however currently there are no validated biomarkers for AD. Recently, several reports suggested that autoantibodies may play role in AD and can be used as diagnostic/prognostic biomarkers for AD as well as understanding AD etiology. In the present work, authors conducted mass spectrometry-based discovery proteomics to identify autoantibodies in CSF of AD subjects that may be used as potential diagnostic markers. The manuscript is well-written and the work is interesting, however following are a few suggestions that will improve the manuscript:

1. Authors should provide additional details on Tissue protein extraction such as which enzyme was used to digest pulverized tissue (as RapiGest SF, a mild denaturant, only solubilizes and unfolds the proteins) "OR" authors meant that RapiGest SF was used to solubilize proteins?
2. Immunoprecipitation: did authors optimize the immunoprecipitation method to minimize binding of non-specific proteins as in the "Data Analysis" section they describe several non-specific proteins were detected (Authors did remove these from the final analyses and that may potentially introduce systematic error/bias)?
3. Data Analysis: authors should provide additional details such as how the peptides/proteins were filtered (e.g., use of >2 unique peptides; and also provide details if they have calculated Log2 fold changes in protein expression between groups). It is reported that ~1 unique peptide per protein is detected and that is not as robust data to confidently detect, identify, and quantify unique proteins.

Is the work clearly and accurately presented and does it cite the current literature?

Yes

If applicable, is the statistical analysis and its interpretation appropriate?

Partly

Are all the source data underlying the results available to ensure full reproducibility?

Yes

Is the study design appropriate and is the work technically sound?

Yes

Are the conclusions drawn adequately supported by the results?

Yes

Are sufficient details of methods and analysis provided to allow replication by others?

Partly

Reviewer Expertise:

Systems Proteomics; Mass Spectrometry-based biomarker discovery and validation; Toxicoproteomics; Secretomics; Host-Pathogen Biology.

I confirm that I have read this submission and believe that I have an appropriate level of expertise to confirm that it is of an acceptable scientific standard, however I have significant reservations, as outlined above.

# Peer review: Reviewer response for version 1

**DOI:** 10.5256/f1000research.23271.r58125

**Published:** 2020-02-07

**Authors:** Stefan Holdenrieder

**Affiliations:** 1 German Heart Centre, Technical University of Munich (TUM), Munich, Germany

The authors present a well-written manuscript on a novel approach for exploring novel autoantibody (AAB) biomarkers in the cerebrospinal fluid (CSF) of patients with Alzheimer’s disease (AD) and Parkinson’s disease (PD). They used an unsupervised mass spectrometry method to identify brain antigens that were bound to AABs in the CSF of these patients but not in headache patients as controls.

 Thereby they identified several novel candidate markers particularly in AD patients that were present in at least half of AD patients but not or almost not in controls. In PD patients, no relevant marker was identified using these criteria. Generally, this is an interesting approach as it overcomes the targeted hypothesis-based search by immunological methods and leads to the detection of until now unknown markers. For some of these new markers, a link to disease pathophysiology was proposed.

 However, the authors also emphasize that this is only the first step of novel marker detection strategy and recognize the limitations of their study, e.g. the low patient number, the presence of confounding factors and the need of validation in a larger cohort by orthogonal methods. Finally basic science is required to establish a link of all novel markers with disease pathogenesis and progression.

 In addition, there may be some more general aspects that should be addressed in the discussion section, e.g. the limited sensitivity of the method for less frequent antigens, the importance of appropriate preparation of the brain tissue templates (were dopaminergic structures of the striatum included?) and the variable antibody specificity for interindividually different antigens on brain structures.

 Furthermore, the heterogeneity of these diseases and the different stages included in the cohort should be discussed. Most importantly, it may be relevant that also other types of dementia (e.g. cerebrovascular) and neuroinflammatory diseases are included as further control groups.

 Regarding the present study it could be discussed whether markers that are present in AD but not in PD (and vice versa) are more relevant as they may be more disease specific. This means that also the other diseased patients could serve as control group. Finally, if then an AAB is present in less than 50% e.g. of PD patients but in none of all other control cohorts, this may be a valuable disease-specific marker although it is not as frequent in the specified disease as other ones.

Is the work clearly and accurately presented and does it cite the current literature?

Yes

If applicable, is the statistical analysis and its interpretation appropriate?

Yes

Are all the source data underlying the results available to ensure full reproducibility?

Yes

Is the study design appropriate and is the work technically sound?

Yes

Are the conclusions drawn adequately supported by the results?

Yes

Are sufficient details of methods and analysis provided to allow replication by others?

Yes

Reviewer Expertise:

NA

I confirm that I have read this submission and believe that I have an appropriate level of expertise to confirm that it is of an acceptable scientific standard, however I have significant reservations, as outlined above.

# Peer review: Reviewer response for version 1

**DOI:** 10.5256/f1000research.23271.r58124

**Published:** 2020-02-05

**Authors:** Ronald A. Booth

**Affiliations:** 1 University of Ottawa, Ottawa, ON, Canada; 2 The Ottawa Hospital, Ottawa, ON, Canada; 3 Eastern Ontario Regional Laboratory Association (EORLA), Ottawa, ON, Canada

The authors present a manuscript detailing a novel mass spectrometric method of identifying putative autoantibodies in CSF.

 Overall, the manuscript is clear and well written. It is however a very preliminary report for the identification of potential autoantibodies, and the authors have addressed this in their limitations. One potentially significant confounding element is the age difference between controls and patients. The AD and PD groups are significantly older, which has the potential to produce false positive autoantibodies, as it is well known that older healthy individuals have a higher rate of autoantibodies in the blood, and presumably in the CSF. The increased rate of autoantibodies may account for the unique autoantibodies found in the PD and AD groups. Use of a control group of a similar age would be preferred.

 I have a few specific comments/questions:

- The authors included a positive control antibody, human kallikrein 6, at a concentration of 100 ng. The concentration used may be supraphysiological and hence create an inappropriate positive control. Have the authors titrated the control antibody concentration to determine the lower limit of detection?  
- In regards to the putative autoantibodies identified in AD patients, have any of the antibodies been confirmed using an alternate technique? ELISA or immunohistochemistry? Without corroboration with an alternate methodology, these may all be false positive results.

Is the work clearly and accurately presented and does it cite the current literature?

Yes

If applicable, is the statistical analysis and its interpretation appropriate?

Yes

Are all the source data underlying the results available to ensure full reproducibility?

Yes

Is the study design appropriate and is the work technically sound?

Partly

Are the conclusions drawn adequately supported by the results?

Yes

Are sufficient details of methods and analysis provided to allow replication by others?

Yes

Reviewer Expertise:

My work is in the area of clinical autoimmune diagnostics.

I confirm that I have read this submission and believe that I have an appropriate level of expertise to confirm that it is of an acceptable scientific standard, however I have significant reservations, as outlined above.

# Peer review: Reviewer response for version 1

**DOI:** 10.5256/f1000research.23271.r58126

**Published:** 2020-01-29

**Authors:** Kiang-Teck Jerry Yeo, Xin Ye

**Affiliations:** 1 Department of Pathology & Section of Clinical Chemistry, The University of Chicago, Chicago, IL, USA; 2 Clinical Pathology & Genomic Medicine, Houston Methodist & Weill Cornell Medical College, Houston, TX, USA

Alzheimer’s disease (AD) and Parkinson’s disease (PD) are two most common neurodegenerative diseases responsible for dementia. Currently, AD is often diagnosed in the mild dementia stage by a combination of clinical tests and brain imaging studies. Numerous studies have highlighted the use of CSF or blood biomarkers in identifying and diagnosing AD at earlier stages, even in the pre-symptomatic phase. Despite the hallmark findings of accumulation of amyloid-β protein (Aβ) and hyper-phosphorylated tau proteins in AD, the pathogenic mechanism that led to the onset and development of disease are still largely unknown. There is increasing evidence suggesting that autoimmune diseases are linked to increased risk of dementia development. Autoantibodies against a variety of antigens were found to be associated with AD, such as autoantibodies to Aβ, tau proteins, neurotransmitters, glial markers, and lipid molecules. There is still a real unmet need to discover sensitive and specific biomarkers for AD and PD, ideally in less invasive, blood samples. Recently, ELISA and microarray based analysis has been applied in an attempt to develop diagnostic tests for AD. 

 The authors described a novel immunoaffinity-based mass spectrometry method to identify potential autoantibody biomarkers in AD and PD. The study limitations, including significant younger age of control group, small sample size (10 in each group), and unknown method sensitivity are discussed. CSF and brain tissue preparation were analyzed using NanoLC-MS, and peptide search are all described in detail. The preset selection criteria for representative autoantibodies in the disease group attempts to identify relatively more disease-specific biomarkers. Overall this study, although small in sample size, is scientifically sound, and presents a good and interesting proteomics approach for biomarker discovery, especially immunoglobulin biomarker study. Several questions are raised by the reviewers regarding this work that may help clarify some aspects of the methods:  

1. Are AD or PD patients truly able to give “informed consent”?
2. How do you convince patients with HA to undergo LP for CSF collection, or is this part of the diagnostic workup?
3. Why was RapiGest SF surfactant used on brain tissue before incubation with bead-bound IgG? RapiGest is a mild denaturant, which unfolds proteins and makes proteins more susceptible to enzymatic cleavage. Most immunoaffinity-based mass spectrometry methods denature proteins after immunoaffinity precipitation and elution. 
4. The authors stated measuring protein concentration of extracted brain tissue and applied an equal amount of tissue protein in each sample. However, did the authors also measure CSF protein and CSF IgG concentration? Were equal amounts of CSF IgG applied in each sample? If not, will low CSF IgG concentration in one sample/or one group result in lower sensitivity on autoantibody detection?   

Is the work clearly and accurately presented and does it cite the current literature?

Yes

If applicable, is the statistical analysis and its interpretation appropriate?

Yes

Are all the source data underlying the results available to ensure full reproducibility?

Yes

Is the study design appropriate and is the work technically sound?

Yes

Are the conclusions drawn adequately supported by the results?

Yes

Are sufficient details of methods and analysis provided to allow replication by others?

Yes

Reviewer Expertise:

Our areas of research encompass blood, CSF, urine, other body fluids biomarkers employed in the diagnosis, prognosis and treatment of diseases.

We confirm that we have read this submission and believe that we have an appropriate level of expertise to confirm that it is of an acceptable scientific standard.

## References

[1] : Exploring Biomarkers for Alzheimer's Disease. *J Clin Diagn Res*.2016;10(7) : 10.7860/JCDR/2016/18828.8166 KE01-6 10.7860/JCDR/2016/18828.8166 27630867PMC5020308

[2] : Autoantibodies in Alzheimer's disease: potential biomarkers, pathogenic roles, and therapeutic implications. *J Biomed Res*.2016;30(5) : 10.7555/JBR.30.20150131 361-372 10.7555/JBR.30.20150131 27476881PMC5044708
